# Thorn-like TiO_2_ nanoarrays with broad spectrum antimicrobial activity through physical puncture and photocatalytic action

**DOI:** 10.1038/s41598-019-50116-0

**Published:** 2019-09-23

**Authors:** Eun-Ju Kim, Mingi Choi, Hyeon Yeong Park, Ji Young Hwang, Hyung-Eun Kim, Seok Won Hong, Jaesang Lee, Kijung Yong, Wooyul Kim

**Affiliations:** 1 Center for Water Resources Cycle Research, Korea Institute of Science and Technology (KIST), Seoul, 02792 Korea; 2 Department of Chemical Engineering, Pohang University of Science and Technology (POSTECH), Pohang, 37673 Korea; 3 Civil, Environmental, and Architectural Engineering, Korea University, Seoul, 02841 Korea; 4 Department of Chemical and Biological Engineering, Sookmyung Women’s University, Seoul, 04310 Korea; 5 Institute of Advanced Materials and Systems, Sookmyung Women’s University, Seoul, 04310 Korea

**Keywords:** Pollution remediation, Pollution remediation, Photocatalysis, Photocatalysis

## Abstract

To overcome the conventional limitation of TiO_2_ disinfection being ineffective under light-free conditions, TiO_2_ nanowire films (TNWs) were prepared and applied to bacterial disinfection under dark and UV illumination. TNW exhibited much higher antibacterial efficiencies against *Escherichia coli* (*E. coli*) under dark and UV illumination conditions compared to TiO_2_ nanoparticle film (TNP) which was almost inactive in the dark, highlighting the additional contribution of the physical interaction between bacterial membrane and NWs. Such a physical contact-based antibacterial activity was related to the NW geometry such as diameter, length, and density. The combined role of physical puncture and photocatalytic action in the mechanism underlying higher bactericidal effect of TNW was systematically examined by TEM, SEM, FTIR, XPS, and potassium ion release analyses. Moreover, TNW revealed antimicrobial activities in a broad spectrum of microorganisms including *Staphylococcus aureus* and MS2 bacteriophage, antibiofilm properties, and good material stability. Overall, we expect that the free-standing and antimicrobial TNW is a promising agent for water disinfection and biomedical applications in the dark and/or UV illumination.

## Introduction

The photocatalytic destruction of microorganisms by TiO_2_ has been well documented over the last several decades^1–4^. However, TiO_2_ is functional only in the presence of UV light, which limits its practical applications in the dark or under visible light. A number of engineering approaches have been adopted to enhance the solar energy utilization of TiO_2_, including doping^5,6^, metallization^7^, and the use of heterojunctions^8,9^. In contrast, there is a paucity of fundamental studies aimed at revealing the activity of TiO_2_ under complete darkness. Notably, several types of metal composites (*i.e*., Ag-, Cu-TiO_2_) exhibit significant antimicrobial capability under dark conditions^10,11^. The antimicrobial mechanism of such materials is mainly attributed to the release of their respective ions, which interact with DNA and proteins and cause cell death. Unfortunately, this raises concerns about the release of toxic metal ions causing adverse health effects and posing ecological risks. Therefore, it is highly desirable to seek a strategy for achieving antimicrobial activities in the dark, while minimizing the release of potentially toxic metal ions.

Nanomaterials with varying morphologies interact differently with cell membranes and thus exhibit a broad spectrum of antimicrobial action, which is associated with the generation of reactive oxygen species (ROS) or fragmentation of the cell membrane^12^. This strong shape-dependent antibacterial activity is well studied for diverse types of nanomaterials like TiO_2_^13^, ZnO^14^, and Ag^15^. Of these, one-dimensional (1D) nanomaterials show a unique mode of cellular interaction called frustrated phagocytosis which pierces the membrane upon contact, thereby injuring or lethally inactivating bacterial cells^16–18^. Recent studies have also demonstrated the remarkable antibacterial activity of 1D nanostructured arrays *via* physical contact killing^19^. The interaction of 1D nanoarrays with the cell membrane depends on cell type and nanostructure properties such as shape, aspect ratio, and density^20–23^. It was shown that surface with high aspect ratio nanostructures provided higher bactericidal effect than the surface with lower aspect ratio nanostructures^21,24^. In this respect, nanowire array that has high aspect ratio topography and large density is a good candidate for this application. Although the antibacterial activity of titania nanowires has been already reported, little data is available in the literature regarding how the surface topography affects their efficiency. Moreover, the coupled role of physical and photochemical actions on the antimicrobial mechanism of titania nanowires under illumination remains limited.

In this study, we highlight the importance of morphological control of titania nanoarrays (nanowire vs nanoparticle) when applying them in disinfection and photocatalytic degradation. The vertical TiO_2_ nanowire film (TNW) exhibit lower photocatalytic activity for the degradation of organic pollutants due to its lower surface area, but enhanced antibacterial activity compared with flat TiO_2_ nanoparticle film (TNP). To explain the enhanced antibacterial activity of TNW, the contribution of physical damage to outer membrane is studied in topography-dependent manner. Additionally, investigations on the reusability and broad-spectrum antimicrobial activity demonstrate that the TNW offers great potential in developing sustainable antimicrobial materials.

## Results and Discussion

### Characterization of the TiO_2_ nanowire film (TNW)

**Figure 1:**
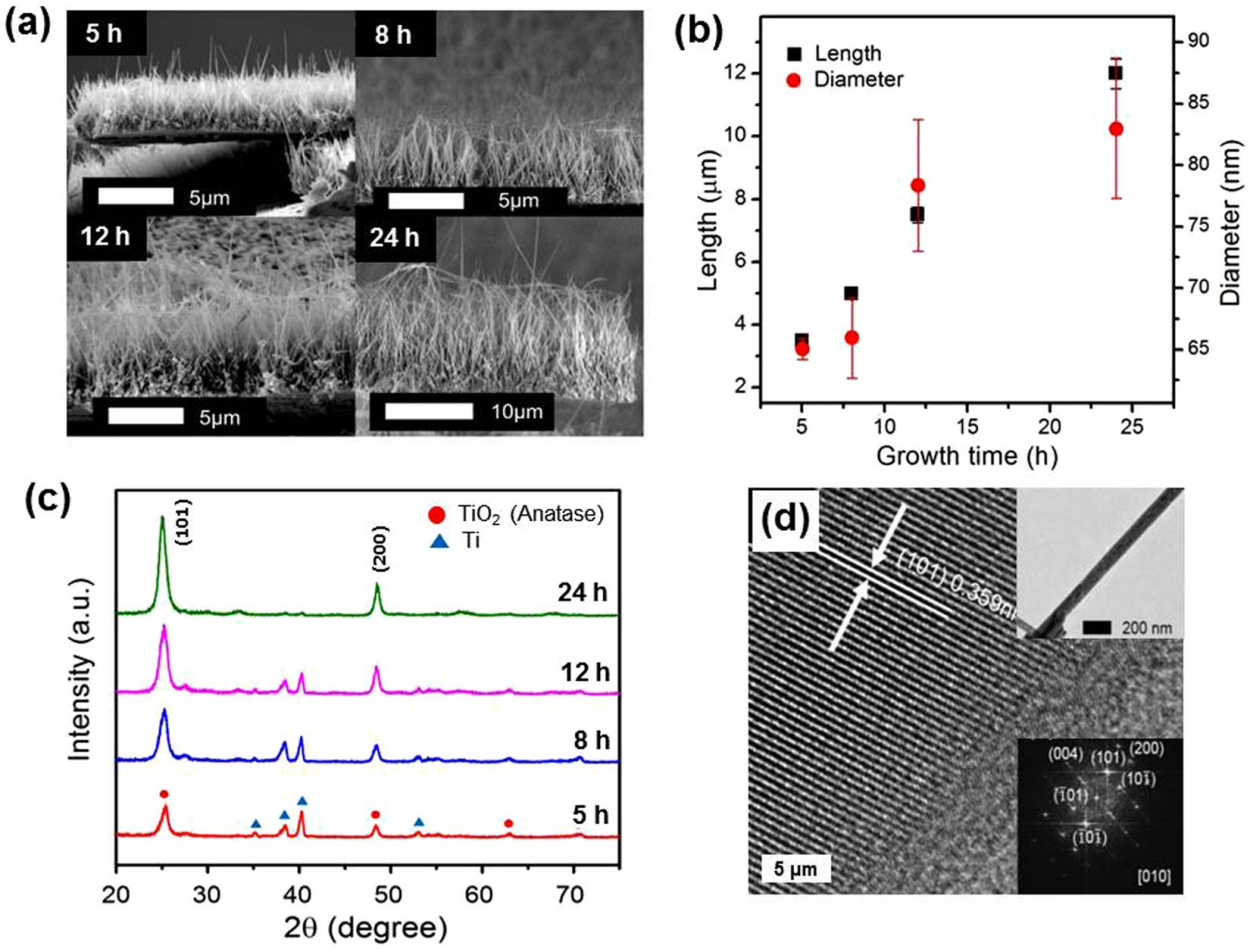
(**a**) Cross-sectional SEM images of TNWs grown for 5, 8, 12, and 24 h. (**b**) NW lengths and diameters were plotted as a function of growth time. (**c**) XRD patterns of TNWs. (**d**) FFT pattern and HRTEM image of TNWs grown for 8 h.

Figure 1a shows cross-sectional SEM images of TNWs grown for different hydrothermal treatment durations (5–24 h). The growth mechanism of the TNWs on Ti foil has been described previously^25–27^. In the early stages of the reaction, nanosheet structures are formed on the Ti foil in the autoclave reactor. TNWs originate from these nanosheet structures, which are transformed into nanowire structures by splitting to reduce their surface energy^28,29^. Obviously, the vertically aligned NWs were uniformly grown on the Ti substrate and increasing the reaction time caused the NWs to merge at their tips, due to van der Waals forces between them^30^. The mean values of the length, diameter, and density of TNW samples determined from SEM images (Fig. 1a and Figure S1) are summarized in Table S1. The length and diameter of NWs gradually increased with prolonging the hydrothermal reaction time, as shown in Fig. 1b. The average length of the NWs was approximately 3.5, 5.0, 7.5, and 12.0 μm for reaction times of 5, 8, 12 and 24 h, respectively, with diameters calculated to be in the range of 65 to 83 nm. The NW number density increased between 5 and 12 h and then decreased at longer growth time (Table S1), which was caused by NW coalescence. The aspect ratio of TNWs ranged from 53.8 to 144.6 (Table S1).

XPS spectra were measured to confirm the types of chemical species formed for the TNW grown for 8 h. The Ti 2p and O 1 s XPS spectra are provided in Figure S2. Ti 2p_1/2_, Ti 2p_3/2_, and O1s peaks of the TNW occurred at binding energies of 458.6 eV, 464.4 eV, and 529.9 eV, respectively, which correspond to the lattice chemical bonding of TiO_2_^31,32^. The XRD profiles of the TNWs are given in Fig. 1c. All the diffraction peaks at 2θ = 25.3°, 48.1°, 62.8°, and 70.5° could be indexed to the (101), (200), (204), and (220) planes of anatase TiO_2_, respectively except the peaks from the Ti substrate. The enhanced (101) peak intensity and disappearance of the (204) and (220) peaks with increasing growth time indicated a (101) preferred crystallization. A high resolution TEM image and selected area electron diffraction (SAED) pattern (Fig. 1d and inset) of an individual NW revealed that they were single-crystalline with an interplanar distance of 0.35 nm, which corresponds to the (101) plane of anatase TiO_2_. The (101) crystal plane was perpendicular to the NW growth axis [010] direction, which confirms the XRD results (Fig. 1c). The results obtained from the Raman spectrum of TNW grown for 8 h using a 514-nm laser are shown in Figure S3. The sample showed typical Raman peaks of anatase at 143 (E_g_), 196 (E_g_), 395 (B_1g_), 516 (B_1g_ + A_1g_), and 638 cm^−1^ (E_g_)^33,34^, indicating that TNWs are well-crystallized anatase.

### Photocatalytic activity comparison of TNWs toward bacteria and chemical substrates

**Figure 2:**
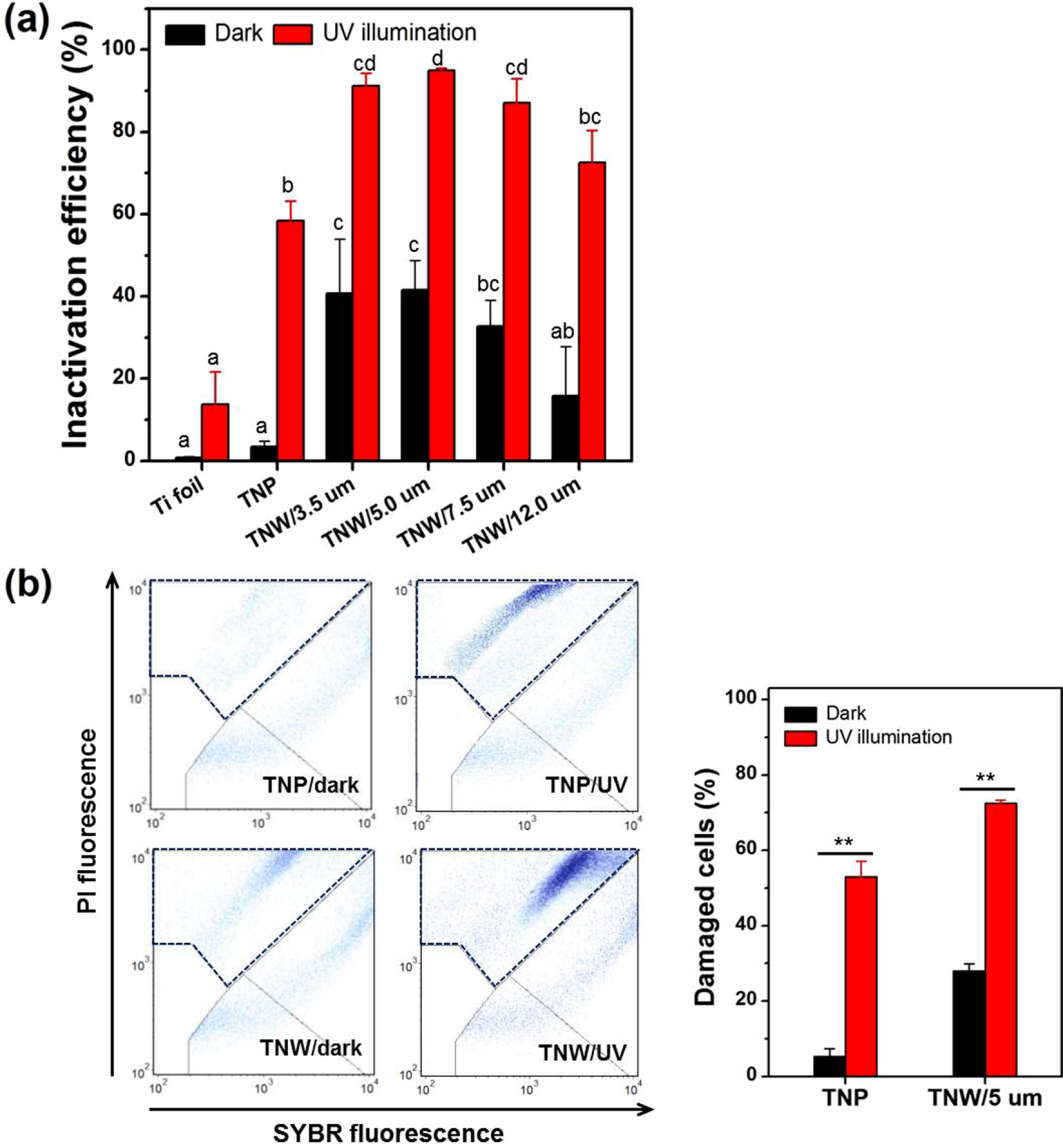
(**a**) Inactivation of *E. coli* by TNP and TNWs with different lengths (3.5, 5.0, 7.5, and 12.0 μm) upon a 30 min exposure determined by plate count method. Different letters above the bars represent significant differences (ANOVA, *P* < 0.05). (**b**) Flow cytometry analysis of *E. coli* after treatment with TNP and TNW/5 μm. The left and right panels in (**b**) display the dot plot and the percentage of damaged cells (indicated by dashed line in dot plot). **P < 0.01, Student’s *t* tests.

The antibacterial activities of the TNWs with different lengths (3.5, 5.0, 7.5, and 12.0 μm) and the TNP in the dark and UV light were determined using plate count method (Fig. 2a). The Gram-negative bacterium *E. coli* was used as a model organism for all inactivation experiments, unless otherwise specified. A slight photocatalytic inactivation efficiency in the Ti foil control sample resulted from the direct action of UV light. The TNWs showed much higher disinfection performance compared with TNP under both reaction conditions (Fig. 2a). In the absence of UV-illumination (dark), considerable bacterial inactivation (ca. 45% for the nanowire length of 5 μm (TNW/5 μm)) occurred in the case of TNW with clear cell piercing, whereas TNP exhibited negligible activity, indicating the physical nature of the bactericidal activity of the NWs. In addition, the higher inactivation efficiencies of TNW compared to TNP under UV light could be ascribed to cell-piercing effect that renders the pierced cells more vulnerable to photo-induced ROS attack. To further confirm this result, the flow cytometric analysis using SYBR/PI staining was applied to reveal changes in membrane integrity after exposure to TNP and TNW/5 µm that displayed the highest antibacterial activity. As depicted in Fig. 2b, the proportion of dead cells (with damaged membranes) in TNW/5 µm was evident in the dark, and higher than that of TNP under UV illumination, which is consistent with the results of the plate counts. The discrepancy in number of viable cells between plate count and flow cytometry is probably due to the presence of viable but non-culturable cells^35^. The bactericidal activity of TNW/5 µm resulting in >30% cell death within 30 min was comparable to that of previously reported nanopillared surfaces against Gram-negative bacteria^20,36–38^, although a straightforward comparison of those results was difficult due to their different surface features and different times of incubation. These results would suggest that cell piercing by association in TNW directly affects membrane integrity and thus cell viability. Clearly, the outer membrane plays a crucial role in survival of bacteria.

With increase in NW length, the efficiencies under dark and UV-illumination decreased to similar extent (ca. 5% for TNW/7.5 µm and ca. 14% for TNW/12 µm based on the efficiency of TNW/5 µm), which supports that the bactericidal effect of TNW is strongly related to a non-ROS-mediated path as well as a typical ROS-mediated path. The physical attack via NW piercing may be highly affected by the physical properties of the NWs (e.g., length, diameter, density and stiffness). Among them, length and stiffness can be considered similar parameters because NW bends when it becomes long. As can be seen from Figure S5, the physical contact-based antibacterial efficiency for TNWs as a function of length, diameter, and density displayed that the efficiency depends on the diameter of NW before NW bending, but depends on the length and surface density of NW as NW exceeds a certain critical length and becomes bent. Therefore, the piercing mechanism of NW cannot be ascribed solely to the single physical property. In cases of longer TNWs, the collision intensity between NWs and bacteria would be lower due to the morphological changes of NWs such as, fusion and bending (see Fig. 1a), thereby resulting in the decrease of inactivation efficacy. For the NW lengths examined herein (3.5–12.0 µm), the optimum length was found to be 5 μm which exhibited the highest the highest bactericidal activity, and TNW/5 µm was chosen for the subsequent experiments.

**Figure 3:**
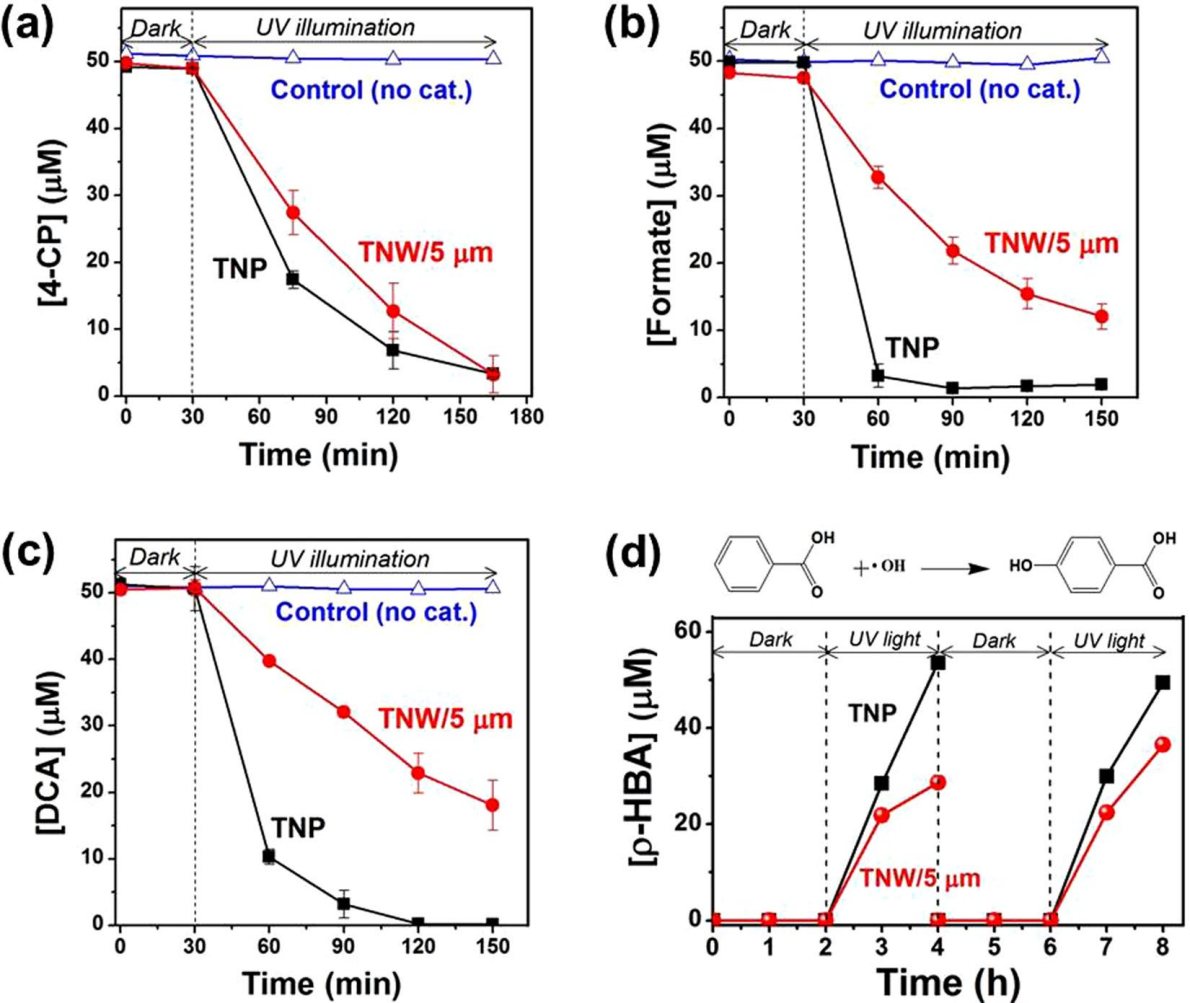
Photocatalytic decomposition of (**a**) 4-CP, (**b**) formate, and (**c**) DCA on TNP and TNW/5 μm. Experimental conditions: [4-CP]_0_ = [DCA]_0_ = [formate]_0_ = 50 μM, pH_i_ = 3.0, and λ > 320 nm. (**d**) Time profiles for the photocatalytic production of *p*-hydroxybenzoic acid (*p*-HBA) from the oxidation of benzoic acid on TNW/5 μm and TNP under successive dark and UV illumination conditions. Experimental conditions: [BA]_0_ = 10 mM, pH_i_ = 3, and λ > 320 nm.

To distinguish between photo-induced ROS-mediated activity and non-ROS-mediated activity on UV-irradiated TNWs for photochemical disinfection, the photocatalytic activities of the TNW/5 µm towards three organic pollutants (4-CP, DCA, and formate) were compared with those of TNP, which is not capable of affecting a physical piercing-type mechanism (Fig. 3a–c). Contrary to the disinfection results, TNP exhibited higher photocatalytic activities than TNW/5 µm, and all chemical substrates showed negligible decomposition in both TNW/5 µm and TNP under dark conditions. Indeed, when the generation of OH radicals was monitored on TNW/5 µm and TNP over repeated photocatalysis using benzoic acid as a chemical trap of ·OH (through reaction in Fig. 3d)^39^, ·OH production was observed only under UV-illumination conditions and not under dark conditions. Moreover, TNP showed better activity for ·OH generation than TNW/5 µm, which is consistent with the photocatalytic degradation results. These results support the idea that non-ROS-mediated activity is not commonly observed in the decomposition of chemical substrates in the dark. Although the activities of TiO_2_ depend on many parameters such as, the sample preparation conditions, properties of reaction medium, and type of substrates, the observed differences between the photocatalytic degradation and disinfection performances may arise from the different sizes (of at least three orders of magnitude) of the chemical substrates and bacteria. The size of chemical substrates is less than the nanometer range but *E. coli* bacterium is approximately 1–2 micrometers long. Since the photocatalytic reaction of chemical substrates mainly occurs on the TiO_2_ surface, the degradation performance preferentially depends on the specific surface area of the TiO_2_ materials. Indeed, TNP had a higher surface area than TNW/5 µm (ca. 4.5 times) based on our N719 dye absorption data (Figure S4). However, Langmuir adsorption of bacteria on TiO_2_ is not possible due to their relatively large size; therefore, it seems that the TiO_2_ morphology has a more appreciable effect on photocatalytic disinfection rather than the surface area. These findings highlighted the importance of morphological modulation in developing strategies to enhance the antibacterial property of pristine TiO_2_, especially in dark environments. In addition, the present study suggests that the assessments of TiO_2_ nanowires for bacterial disinfection are difficult to be generalized to include chemical substrates. This is largely because of not only the vastly different substrate sizes relative to bacteria but also because of the operative non-ROS-mediated disinfection pathway under dark conditions.

### Role of physical puncture in TNW disinfection mechanism under dark and UV illumination

**Figure 4:**
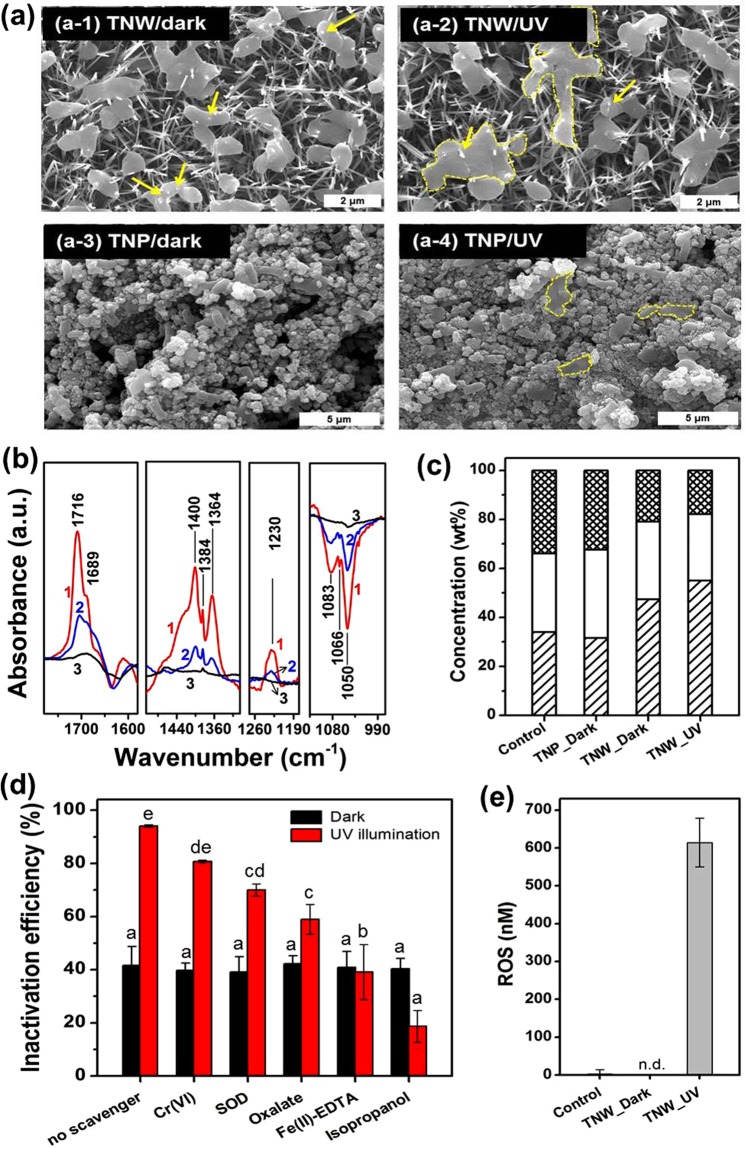
(**a**) ESEM images of *E. coli* after a 30 min treatment with (a-1) TNW/5 μm in the dark, (a-2) TNW/5 μm under UV irradiation, (a-3) TNP in the dark, and (a -4) TNP under UV irradiation. The yellow arrows in the images represent the cell directly penetrated by NWs. Some of the bacteria were flattened and aggregated each other, indicating severe damage (yellow dash lines). (**b**) FT-IR difference spectra between samples and *E. coli* control (trace 1, TNW/5 μm under UV irradiation – *E. coli*; trace 2, TNW/5 μm in the dark – *E. coli*; trace 3, TNP in the dark – *E. coli*). (**c**) Molecular composition (weight percent) of the surface of *E. coli* cells before and after treatment deduced from XPS data. ▩, hydrocarbon compounds; □, polysaccharides; ▨, peptides. (**d**) Inactivation efficiencies toward *E. coli* with TNW/5 μm in the presence of various scavengers. Different letters above the bars represent significant differences (ANOVA, *P* < 0.05). Experimental conditions were [Cr(VI)]_0_ = 0.05 mM, [SOD]_0_ = 70 mg/L, [oxalate]_0_ = 0.5 mM, [Fe(II)-EDTA]_0_ = 0.1 mM, and [isopropanol]_0_ = 0.3 M. (**e**) Extracellular ROS production.

To reveal the disinfection mechanism of the TNWs, TEM was used to identify the morphological changes of *E. coli* when exposed to them (Figure S6). The untreated *E. coli* showed normal morphology and their internal structures remained intact (Figure S6a). In contrast, membrane damage occurred upon exposure to the TNWs under dark conditions, which was evidenced by the formation of large vacuoles within the cytoplasm and presence of pits and cell debris on the surface (Figure S6b). As expected, destruction of the *E. coli* cells was more serious under UV-irradiated TNWs and the cell interior became white/empty due to both detachment of the cell membrane and release of its cytoplasmic components (Figure S6c). Figure 4a presents typical ESEM images in which the interaction of the TNPs or TNWs with the bacterial cells is demonstrated. As shown in Fig. 4a-1,2, approximately 2–3 NWs on average protruded into each cell, with slight distortion of the membrane around the NWs. In marked contrast, cells on the flat TNP were morphologically intact under the same culturing conditions (Fig. 4a-3). After UV irradiation, the cells became more flattened and deformed on both sample surfaces, which was more pronounced for the TNWs (Fig. 4a-2 vs. 4a-4). Although the TNPs show higher photocatalytic activity (seen in Fig. 3), the physical piercing of the bacteria by the NWs is considered to contribute to a crucial synergistic effect with the photocatalytic disinfection process. Based on the TEM and ESEM investigations, upon interaction with the TNW, the cell envelope of *E. coli* is more severely damaged due to the additional contribution of physical stress induced by the NWs, as well as photo-induced ROS from the UV-irradiated TNW.

Although TEM and SEM analyses give a clear but specific (*i.e*., local) indication of membrane damage, the FT-IR spectroscopic technique, which provides molecular-level information on the bulk cell structure and its composition, was also employed as it provides a complementary adjunct toward a better understanding of the bacterial inactivation mechanism. Figure S7 shows infrared bands of *E. coli* treated for 30 min on TNW under UV irradiation (trace 1), TNW under dark (trace 2), TNP under dark (trace 3), and the untreated control (*E. coli* only) (trace 4). All sample wafers were prepared by mixing KBr powder after freeze-drying treatment. Infrared bands of *E. coli*, shown as trace 4 in Figure S7, agreed well with that of the attenuated transmitted reflectance (ATR)-FT-IR spectra of *E. coli* in water (data not shown), indicating that the bacterial structure remained intact even after its loading into the KBr wafer. The FT-IR spectra in the fingerprint region were well assigned to amide I and II at 1654 and 1535 cm^−1^, -CH_2_ scissor vibration at 1469 cm^−1^, nucleic acid around 1280–1200 cm^−1^, and sugar ring vibrations of the outer membrane (including lipopolysaccharides (LPS), exo-polysaccharides (EPS), etc.) in the 1200-1000 cm^−1^ region^40–44^.

In the case of TNW under both dark and UV illumination conditions, the changes in four distinct spectral regions that characterize the major cellular constituents were significant (Fig. 4b): (i) growth of typical infrared bands of *α*, *β*-unsaturated aldehydes and carboxy groups at 1716, 1689, 1400, 1384, and 1364 cm^−1^;^45^ (ii) increase in the PO_2_^−^ bands centered at 1230 cm^−1^, as an indicator for the structural organization of lipids;^46^ and (iii) decrease in polysaccharide bands at approximately 1100-1000 cm^−1^, which represent the contribution of the external bilayer of the asymmetric outer membrane^40,42^. In accordance with the FT-IR results, both photocatalytic and physical attack of the *E. coli* by TNW induced compositional and conformational changes in several components of the cell membrane, resulting in loss of membrane integrity. To distinguish the physical damage in the dark from photocatalytic damage, we compared the spectra obtained for TNW- and TNP-treated cells in the dark. It is noteworthy that the infrared bands of the TNW sample under dark changed exactly in the same position (but to a smaller extent) as those of UV-irradiated TNW (traces 1 and 2 in Fig. 4b), whereas negligible changes in response to TNP exposure were observed (trace 3 in Fig. 4b). These results support the view that physical attack of the *E. coli* by the sharp NWs in the dark was also responsible for a significant portion of bacterial disinfection.

To corroborate the FT-IR data in terms of changes in basic components of the *E. coli* cell surface, the elemental and molecular compositions were characterized before and after treatment by XPS. The O 1 s, N 1 s, C 1 s, and P 2p spectra for untreated and treated cells under different conditions (TNP in the dark, TNW in the dark and UV light) are shown in Figure S8. Even though UV-irradiated TNW caused the most prominent chemical changes (c and d in Figure S8), we noted some similarities in the spectra of TNW samples under dark and UV light: (i) increase in the P 2p_3/2_ peak at 134.5 eV and O 1 s peak at 534.2 eV, (ii) shift of the C-O peak toward higher binding energies in the O 1 s spectra due to the protonation, and (iii) decrease in C-C,H at 285.2 eV and increase in C-O at 286.6 eV in the C 1 s, implying the damage to carbon-rich lipid layer^47^. Moreover, XPS data can be used to determine the relative concentration of main constituents of bacterial outer membrane, namely peptides, polysaccharides, and hydrocarbon-like compounds (referred to as lipids) that are calculated by a set of three Eqs (1–3)^48^.

1 $${[{\rm{N}}/{\rm{C}}]}_{{\rm{o}}{\rm{b}}{\rm{s}}}=0.279\,({{\rm{C}}}_{{\rm{\Pr }}}/{\rm{C}})$$

2 $${[{\rm{O}}/{\rm{C}}]}_{{\rm{o}}{\rm{b}}{\rm{s}}}=0.325\,({{\rm{C}}}_{{\rm{\Pr }}}/{\rm{C}})+0.833\,({{\rm{C}}}_{{\rm{PS}}}/{\rm{C}})$$

3 $$1=({{\rm{C}}}_{{\rm{\Pr }}}/{\rm{C}})+({{\rm{C}}}_{{\rm{PS}}}/{\rm{C}})+({{\rm{C}}}_{{\rm{HC}}}/{\rm{C}})$$

The proportion of carbon associated with peptide (C_Pr_/C), polysaccharide (C_PS_/C), and hydrocarbon-like compounds (C_HC_/C) could be further converted into weight fractions, using the carbon concentration of each constituent^48^. Figure 4c presents the molecular composition the surface of *E. coli* cells before and after treatment calculated from atomic concentration ratios in Table S2. The significant differences between TNW-treated samples and control were observed in the concentrations of hydrocarbon-like compound and peptide: after incubation on TNW under dark and UV illumination, *E. coli* exhibited a lower hydrocarbon-like compound content and a higher peptide content. These marked changes could possibly be signs of partial damage to the surface lipid layer and outer membrane disruption. In contrast, the effect of TNP on the molecular composition was virtually negligible. Collectively, the analyses from microscopy (TEM and SEM), FT-IR spectroscopy, and XPS strongly reveal the existence of physical damage to the bacteria by the NWs under dark conditions.

The observed alterations in the ultrastructure and membrane properties of *E. coli* after treatment with TNW led us to assess the release of cytoplasmic material following membrane disruption. Potassium ion (K^+^), a universally existent component in bacteria, plays roles in the regulation of polysome content and protein synthesis. Therefore, the release of K^+^ ions from *E. coli* cells was monitored, and the results are shown in Figure S9. As seen, there was no significant potassium ion leakage from *E. coli* in the presence of TNP under dark conditions; recall that TNP does not possess the physical piercing capability toward bacteria as the NWs. However, in response to TNW exposure under dark, a substantial amount of K^+^ leaked out and reached a stable value of 1.27 ppm, which further verifies that vertical NWs can directly damage the cell structure via impaling the outer membrane with their sharp protrusions, leading to a change in membrane permeability. After UV light irradiation with TNP and TNW, much higher concentrations of K^+^ were released compared with those of the dark controls, and the difference between the two data sets was very significant (2.06 ppm for TNP vs 3.02 ppm for TNW). The higher K^+^ value for the UV-irradiated TNW is another line of evidence that shows that membrane damage likely occurred due to a combination of both photocatalytic and physical processes.

The bandgap excitation of TiO_2_ creates photogenerated charge carriers (electrons and holes) that are subsequently trapped on the surface or recombine with each other. These trapped electrons (e_trap_^−^) and holes (h_trap_^+^) either directly oxidize bacteria or indirectly generate ROS such as, ·OH, H_2_O_2_, and O2^·−^. Such direct and indirect oxidative stressors are widely considered to comprise the photo-induced antibacterial mechanism of UV-illuminated TiO_2_^3,49^. The possible roles of reactive species including e_trap_^−^, h_trap_^+^, and ROS in the antibacterial mechanism of TNW were investigated by using appropriate scavengers of these species in the presence and absence of UV-irradiation (Fig. 4d). The scavengers used in this study were isopropanol for ·OH, Cr(VI) for e^−^, superoxide dismutase (SOD) for ·O_2_^−^, oxalate for h^+^, and Fe(II) for H_2_O_2_. Because each scavenger interferes with other reactions, the interpretation of the results may not be straightforward. Nevertheless, it was obvious that in the presence of UV light, ·OH was involved as the main active oxidant in this system (Fig. 4d). The primary role of ·OH in photocatalytic disinfection by TiO_2_ has been frequently proposed^50–52^. On the other hand, the inactivation efficiencies were totally unaffected by the addition of any scavengers (Fig. 4d) and there was no ROS generation under dark conditions (Fig. 4e). These results show that the physical puncture of bacteria should be disinfection pathway in the absence of UV light and is not related to conventional ROS-mediated disinfection pathway in the presence of UV light (see Fig. 4e). Overall, the antibacterial activity of TNWs results from the bacterial membrane puncture, changes in membrane structure and permeability, and ROS-mediated oxidative stress through the synergistic physical and photochemical actions.

### Assessment of practical disinfection applicability of TNWs

**Figure 5:**
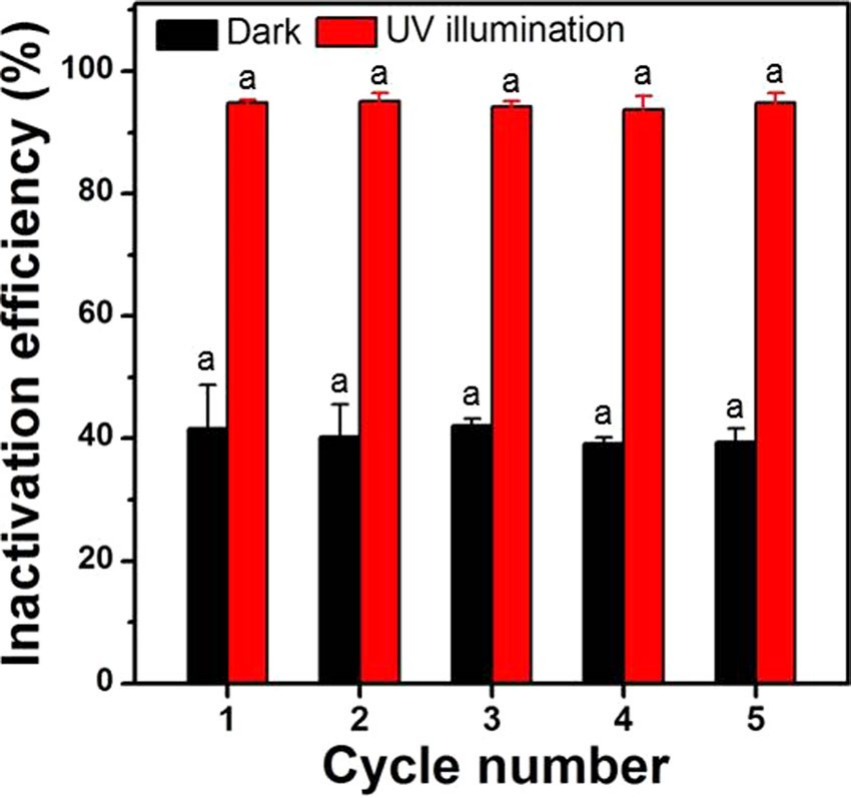
Reusability test of TNW/5 μm up to 5 cycles for the disinfection of *E. coli*. The same letter indicates no significant difference at the 95% confidence level (ANOVA).

**Figure 6:**
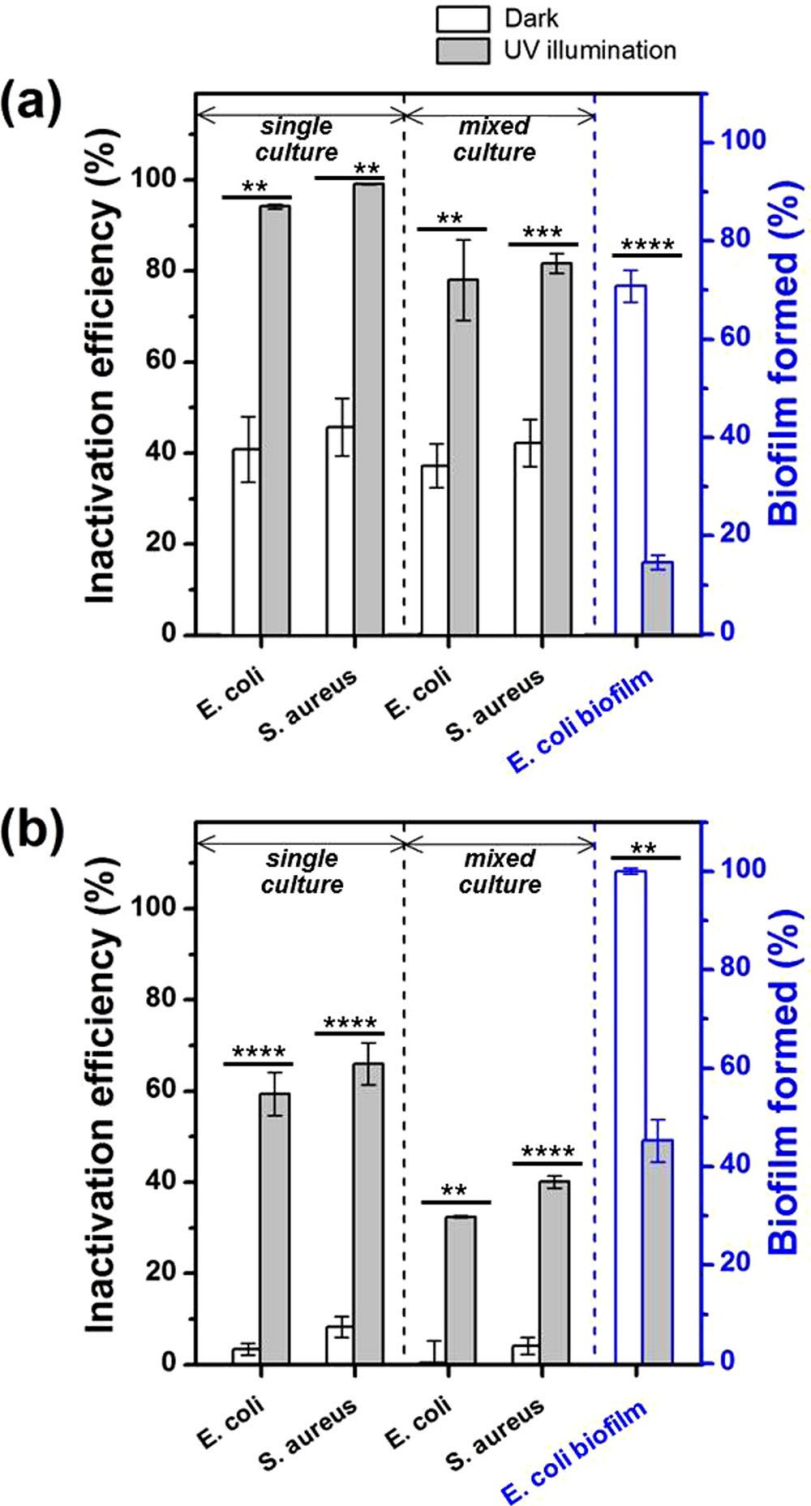
Inactivation of *E. coli* and *S. aureus* as single and mixed cultures and disruption of *E. coli* biofilm in the presence of (**a**) TNW/5 μm and (**b**) TNP with/without UV light for 30 min. Biofilm amount is presented as percentages of the control (the amount of Ti foil in the dark). **P < 0.01, ***P < 0.001, ****P < 0.0001, Student’s *t* tests.

The reusability of TNW for disinfection was evaluated. As depicted in Fig. 5, the antibacterial activity of TNW/5 µm was stable over five repeated cycles in the dark and UV light with no Ti leaching (below 1 ppb as determined by ICP-MS), indicating that TNW/5 µm can be recycled multiple times. Before reuse, the TNW/5 µm surface was cleaned by ethanol and water, and bacterial residuals were not found on the surface after cleaning (Figure S10). For the practical use of TNWs, it is important not only to exhibit stable antibacterial activity, but also to maintain activity in a broad range of microorganisms. Toward this end, we assessed the inactivation efficiency of TNWs against other microorganisms (Gram-positive bacterium *S. aureus* and MS2 bacteriophage) and *E. coli* biofilm (Fig. 6 and Table S3). Notably, TNW displayed higher antimicrobial activities than those of TNP under dark and light conditions, although some variations in the disinfection efficiencies were evident depending on the type of microorganism. In both samples, bacteria were more resistant compared with virus, and the bactericidal efficacy for Gram-positive *S. aureus* was slightly higher than that for Gram-negative *E. coli*. To offset the harmful effect of ROS, many bacteria have evolved antioxidant enzyme systems, which is expected to contribute to lower bacterial death rates. The slightly dissimilar inactivation responses between Gram-positive and Gram-negative bacteria likely arise from structural differences in their cell walls^53^. In addition, TNW could inhibit biofilm formation in the dark and significantly inactivate biofilm cells in the UV light when compared with TNP.

The majority of reported inactivation experiments has been carried out using pure bacterial cultures. However, since bacteria frequently co-exist in natural environments, it is necessary to evaluate the disinfection performances of TNW and TNP against an artificially mixed bacterial culture containing *E. coli* and *S. aureus*. As shown in Fig. 6, the inactivation efficiency of TNW in the mixed culture was almost identical to that of the same strains used separately (as a single culture) under dark, while it decreased slightly under UV light, possibly due to competition between species for the generated ROS. Interestingly, the reduced bacteria inactivation efficiency was much less pronounced in TNW (~10%) compared to that in TNP (~45%), suggesting that apart from the ROS-mediated path, physical penetration caused by the NW morphology plays a role in stabilizing the performance of antibacterial activity in mixed cultures. In other words, this difference in catalyst deactivation is likely to be more substantial in natural environments where diverse microorganisms co-exist in high numbers. Therefore, the role of physical damage is of considerable importance in water disinfection using TiO_2_ photocatalysts.

## Materials and Methods

### Fabrication and characterization of anatase TiO_2_ nanowire films

Anatase TiO_2_ nanowire films (TNW) were prepared by a previously reported hydrothermal method^54^, as detailed in the Supplementary Information. For comparison with the TNW, a TiO_2_ nanoparticle film (TNP) was also fabricated by the conventional doctor blade method using a commercial TiO_2_ powder (Hombikat UV-100, Sachtleben Chemie GmbH), an anatase with a BET surface area of 350 m^2^ g^−1^ and a primary particle size of ~16 nm^55^.

The material characterization included field-emission scanning electron microscopy (FE-SEM), high-resolution transmission electron microscopy (HR-TEM), X-ray diffraction (XRD), X-ray photoelectron spectroscopy (XPS), and Raman spectroscopy. To compare the surface areas of TNP and TNW, the surface area of TiO_2_ nanostructures can be extrapolated by the amount of an adsorbed and desorbed dye^56^. Detailed information can be found in the Supplementary Information.

### Measurement of antimicrobial and photocatalytic activities

The antimicrobial activities of the TNW and TNP against *Escherichia coli* (*E. coli*), *Staphylococcus aureus* (*S. aureus*), and MS2 coliphage were examined using the drop-test method. The cleaned film with ethanol was placed in a sterilized Petri dish, and 100 μL of microorganism suspension (~10^7^ CFU/mL or PFU/mL) was added dropwise to the surface of each sample. The Petri dish was covered with a quartz plate and kept in the dark or UV illumination (a black light blue (BLB) lamp (4-W, λ = 350–400 nm)). After a certain period, microbial inactivation (efficiency (%) = 100 × (1 − *N*_*t*_/*N*_0_)) was calculated by measuring the number of viable cells or viruses before (*N*_0_) and after treatment (*N*_*t*_) using plate count method or plaque assay. The biofilm formation was evaluated by a method described earlier^57^, with slight modifications. *E*. *coli* cells (10^6^ CFU/mL) were incubated with the TNW and TNP for 48 h. The film was removed from the reactor, followed by gently ringing with PBS. The film was then placed in a sterilized Petri dish and kept in the dark or UV illumination. After the reaction, the biofilm cells were detached from the film by sonication for 5 min in 10 mL PBS solution. Biofilm amounts were calculated by plate count method and presented as percentages of control (the amount of Ti foil in the dark).

The photocatalytic activities of the TNW and TNP were studied by monitoring the decompositions of 4-CP, DCA, and formate. A 300-W Xe arc lamp (Oriel) combined with a 320 nm cut-off filter was used as the light source. In brief, the films were placed into 3 mL of 4-CP, DCA, and formate solutions (50 μM) in a cuvette cell. Prior to the photocatalytic reaction, the sample was allowed to reach adsorption/desorption equilibrium for 30 min. The concentration of 4-CP was quantified using high-performance liquid chromatography (HPLC; LC-20A Prominence series, Shimazu). The eluent consisted of a 0.1% phosphoric acid aqueous solution and acetonitrile (80:20, v/v). The analyses of DCA and formate were performed using an ion chromatograph (IC; Dionex DX-120, Thermo Scientific) equipped with a Dionex IonPac AS 14 (4 mm × 250 mm) column for anions and a conductivity detector. The eluent solutions were Na_2_CO_3_ (3.5 mM)/NaHCO_3_ (1 mM).

### Flow cytometry

*E. coli* before and after treatment with TNP and TNW were fluorescently stained with a mixture of SYBR Green I and propidium iodide (PI). After incubation at 36 °C for 15 min in the dark, flow cytometeric analysis was performed by using a Partec Cube 6 flow cytometer (Partec Gmbh).

### Bacterial characterizations

For TEM observation, *E. coli* before and after treatment with TNW was collected, fixed with 2.5% glutaraldehyde and 1% osmium tetroxide, dehydrated in ethanol, and embedded in Epoxy resin and propylene oxide before being sectioned by an ultra-tome. TEM images were obtained using a FEI Tecnai F20 TEM. Cells cultured on the TNW and TNP were air dried, sputter-coated with Pd/Pt for 20 s, and then inserted into a FEI XL-30 environmental SEM (ESEM). FT-IR spectra of treated *E. coli* (in solid form) were collected using FT-IR spectroscopy (Bruker model 80 V equipped with an LN_2_ cooled MCT detector). XPS samples were prepared by dropping bacterial suspensions onto the cleaned silicon wafer and freeze-dried. XPS spectra were recorded on a VG-ESCALAB 250 spectrometer with A1 Kα X-rays as photon source (1486.6 eV).

### Potassium ion (K^+^) release

To investigate K^+^ leakage from *E. coli* during the disinfection process, cell suspensions under different conditions were collected at regular time intervals and then filtered through a 0.45 μm Millipore filter. The analysis of K^+^ was performed using an ion chromatograph (IC, Dionex DX-120).

### ROS generation

The ROS concentration in the cell suspension was quantified with OxiSelect *in vitro* ROS/reactive nitrogen species (RNS) assay kit (catalog number STA-347; Cell Biolabs. Inc.), according to the manufacturer’s instruction. The fluorescence was measured at 480 nm excitation/530 nm emission on a fluorescent plate reader.

### Statistical analysis

Each experiment was conducted at least three times. One-way analysis of variance (ANOVA) with Tukey HSD test (IBM SPSS statistics 20) or Student’s *t*-test (Microsoft Excel) was conducted to assess statistical differences between multiple groups or between two groups, respectively. Data were presented as mean ± standard deviation (S.D.) and probability (*P*) values < 0.05 or 0.01 were considered statistically significant.

## Supplementary information

Supplementary information
