# Supplementary material for: Thorn-like TiO2 nanoarrays with broad spectrum antimicrobial activity through physical puncture and photocatalytic action
Source: Sci Rep. 2019 Sep 23;9:13697. doi: 10.1038/s41598-019-50116-0 (PMC6757029; doi:10.1038/s41598-019-50116-0)
Supplement: Supplementary file 1 — Supplementary information [file 41598_2019_50116_MOESM1_ESM.docx]

Supplementary Information

**Thorn-like TiO_2_ nanoarrays with broad spectrum antimicrobial activity through physical puncture and photocatalytic action**

Eun-Ju Kim^1,†^, Mingi Choi^2,†^, Hyeon Yeong Park^1,3^, Ji Young Hwang^4^, Hyung-Eun Kim^1^, Seok Won Hong^1^, Jaesang Lee^3^, Kijung Yong^2,^* & Wooyul Kim^4, 5,^*

(^†^These authors contributed equally to this work.)

^1^Center for Water Resources Cycle Research, Korea Institute of Science and Technology (KIST), Seoul 02792, Korea

^2^Department of Chemical Engineering, Pohang University of Science and Technology (POSTECH), Pohang 37673, Korea

^3^Civil, Environmental, and Architectural Engineering, Korea University, Seoul 02841, Korea

**^4^**Department of Chemical and Biological Engineering, ^5^Institute of Advanced Materials and Systems, Sookmyung Women’s University, Seoul 04310, Korea

*Corresponding author: kyong@postech.ac.kr, wykim@sookmyung.ac.kr

**Materials and chemicals**

The following reagents were obtained from Sigma-Aldrich and used as received: 4-chlorophenol (4-CP), dichloroacetate (DCA, CHCl_2_CO_2_Na), formate, benzoic acid, acetonitrile, hexavalent chromium, (Cr(VI), Na_2_Cr_2_O_7_∙2H_2_O), superoxide dismutase (SOD, manganese-containing enzyme, lyophilized), oxalate, Fe(II)-EDTA, isopropanol, titanium foil (0.127 mm thick, 99.7% trace metals basis), sodium hydroxide (NaOH, pellets, semiconductor grade, 99.99% trace metals basis), hydrochloric acid (HCl, ACS reagent, 37%), ethanol, and HClO_4_ (ACS Reagent, 70%). HClO_4_ and NaOH were used to adjust the pH of the aqueous suspensions. *Escherichia coli* (*E. coli*, KCTC 2571) and *Staphylococcus aureus* (*S. aureus*, KCTC 3881) were obtained from the Korean Collection for Type Culture (KCTC). MS2 bacteriophage (ATCC 15597-B1) was purchased from the American Tissue Type Culture Collection (ATCC). All working solutions were prepared with deionized ultrapure water (18 MΩ∙cm), which was obtained by a Barnstead purification system.

**Preparation of anatase TiO_2_ nanowire films (TNWs)**

A clean Ti foil (3 × 5 cm^2^) was immersed with the conducting side face down in 0.5 M NaOH and reacted in a Teflon-lined stainless-steel autoclave. The reactor was placed in an oven at 220 ºC for different times (5−24 h) to control the NW length. After the reactor was cooled to room temperature for 2 h, the sample was immersed in 1 M HCl for 10 min. Thereafter, the samples were rinsed with water and ethanol, and then annealed at 500 ºC for 3 h in air.

**Characterization**

The morphology of the TNWs was observed by field-emission scanning electron microscopy (FE-SEM; XL30S, Philips) operating with a 5.0-kV beam energy and high-resolution scanning transmission electron microscopy (HR-STEM; JEM-2200FS with Image Cs-corrector; JEOL) operating with a 200-kV beam energy. The fast-Fourier transformation (FFT) patterns from TEM, X-ray diffraction (XRD; D/MAX-2500, Rigaku) with Cu Kα radiation (40 kV, 100 mA), and Raman spectroscopy (Alpha300R, WITec) with an excitation wavelength of 514 nm were used to verify the crystal structures. X-ray photoelectron spectroscopy (XPS, ESCA LAB 250, VG scientific) using 15 kV monochromatic Al-Kα radiation over an analysis area of 1.1 mm × 1.1 mm was used to determine the surface composition, and the results were calibrated at C 1s peak (285 eV). The surface areas of TiO_2_ nanostructures can be extrapolated by the amount of an adsorbed and desorbed dye. In this case, the TNP and TNW samples were immersed in a 0.3 mM N719 (C_58_H_86_O_8_N_8_S_2_Ru) solution for 24 h. After complete adsorption, the samples were rinsed by ethanol and then immersed in a 0.1 M NaOH solution for desorption. The concentration of the desorbed dye solution was determined spectrophotometrically by monitoring its absorbance.

***
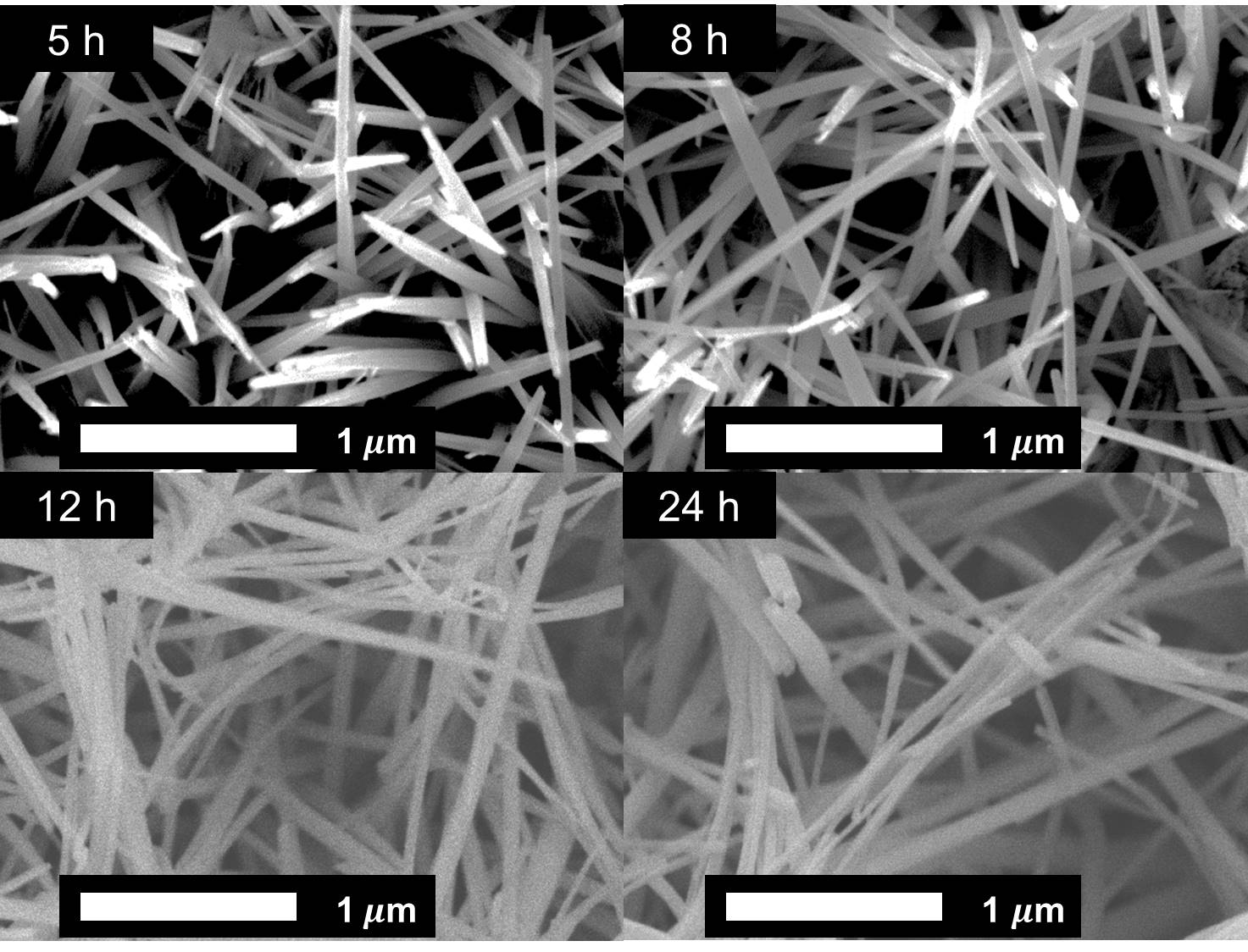
***

**Figure S1.** Top-view SEM images of TNW samples used to determine the diameter and density using ImageJ software.

**Table S1.** Average length, diameter, density, and aspect ratio (length/diameter) of TNW samples at different growth times

| Growth time | Length  (μm) | Diameter  (nm) | Density  (N/μm^2^) | Aspect ratio |
| --- | --- | --- | --- | --- |
| 5 h | 3.5 ± 0.1 | 65 ± 1 | 22.7 ± 2.1 | 53.8 |
| 8 h | 5.0 ± 0.2 | 66 ± 3 | 26.3 ± 1.5 | 75.8 |
| 12 h | 7.5 ± 0.3 | 78 ± 5 | 21.3 ± 3.0 | 96.2 |
| 24 h | 12.0 ± 0.5 | 83 ± 6 | 15.7 ± 2.1 | 144.6 |

**Figure S2.** (a) Ti 2p and (b) O 1s XPS analysis of TNW/5 μm using 15 kV monochromatic Al X-rays.

**Figure S3.** Raman spectrum of TNW/5 μm using a 514-nm laser.

**

**

**Figure S4.** UV-Vis absorption spectra of the desorbed N-719 dye solution from TNP and TNW/5 μm.

**
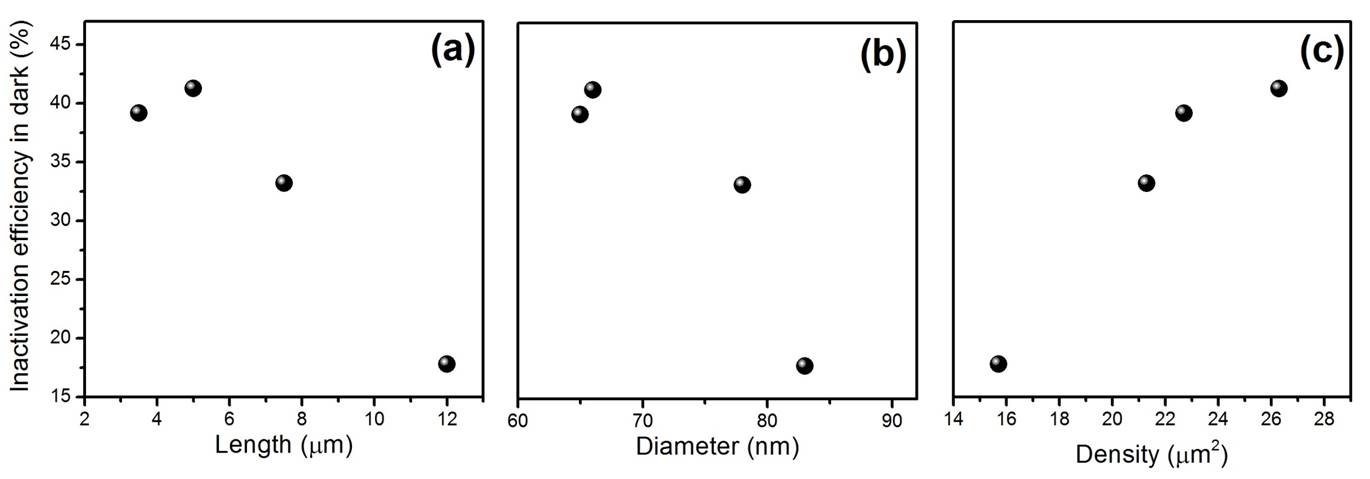
**

**Figure S5.** *E. coli* inactivation efficiency of TNWs in dark as a function of (a) length, (b) diameter, and (c) number density of NWs.

**
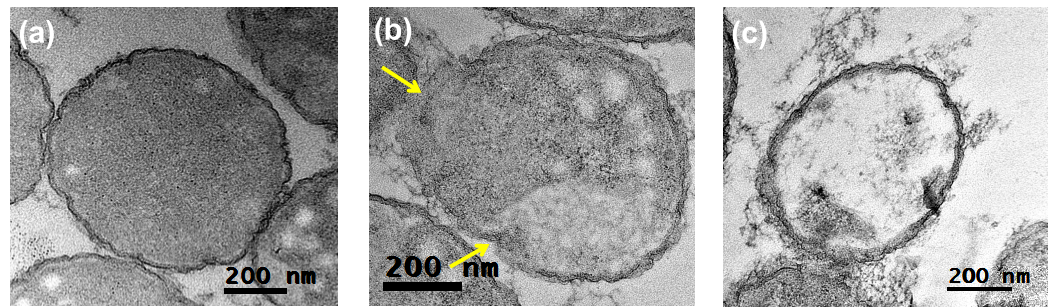
**

**Figure S6.** TEM images of *E. coli* (a) untreated and treated with TNW/5 μm (b) in the dark and (c) under UV irradiation.

**
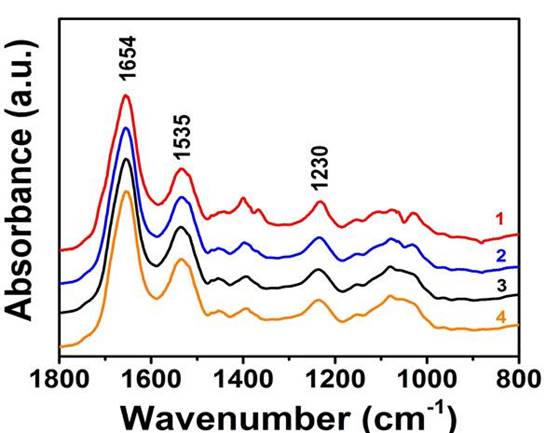
**

**Figure S7.** Enlarged FT-IR spectra of *E. coli* exposed to TNW/5 μm and TNP under different conditions (trace 1, TNW/5 μm under UV irradiation; trace 2, TNW/5 μm in the dark; trace 3, TNP in the dark; trace 4, *E. coli* only).

**
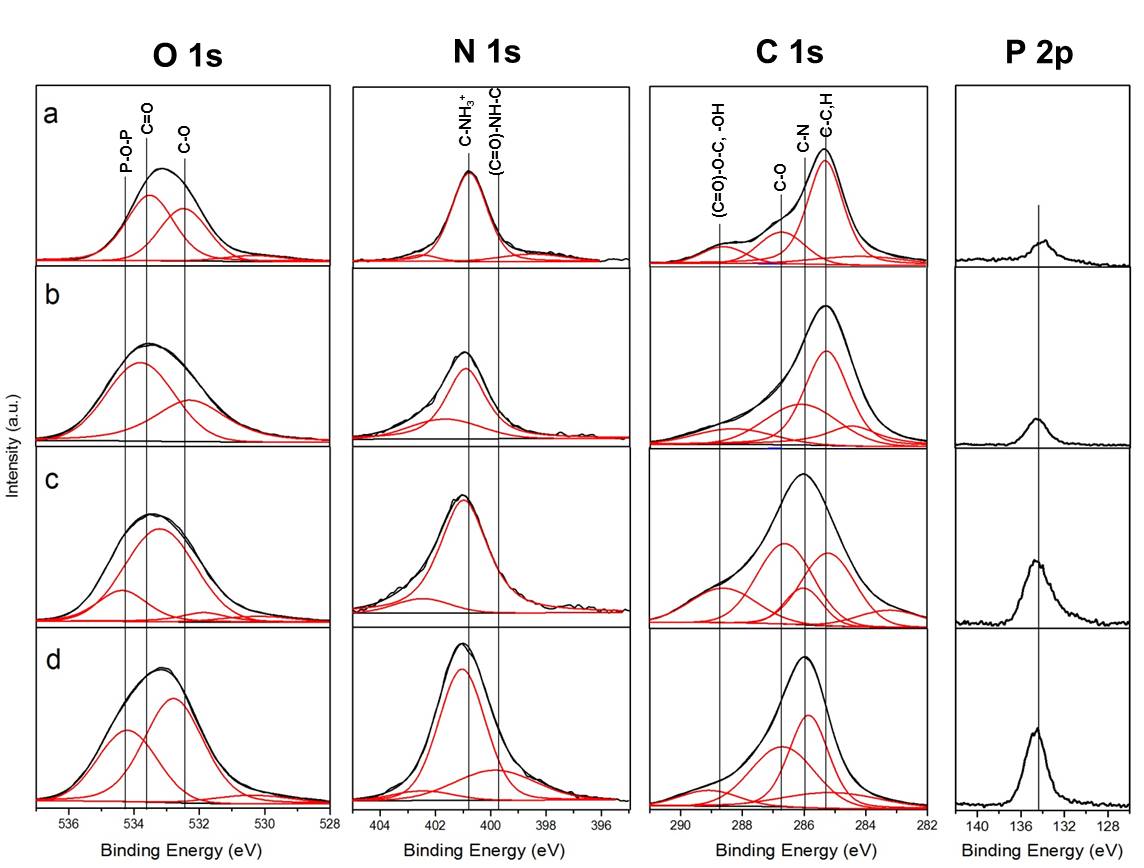
**

**Figure S8.** High resolution O 1s, N 1s, C 1s and P 2p XPS spectra of *E. coli* exposed to TNW/5 μm and TNP under different conditions (a, *E. coli* only; b, TNP in the dark; c, TNW/5 μm in the dark; d, TNW/5 μm under UV irradiation). Peak assignments are indicated in the figure.

**Table S2.** Surface composition determined by XPS*^a^*

| Sample | O/C | N/C |
| --- | --- | --- |
| *E. coli* control | 27.8 ± 0.4 | 7.7 ± 0.1 |
| *E. coli* under TNP/dark | 29.5 ± 0.2 | 7.2 ± 0.1 |
| *E. coli* under TNW/dark | 31.2 ± 0.7 | 10.8 ± 0.2 |
| *E. coli* under TNW/UV light | 30.5 ± 0.1 | 12.5 ± 0.0 |

*^a^* Data are expressed as atomic concentration ratios with respect to total carbon, multiplied by 100. Values represent means±s.d.s for two sets of determination.

**

**

**Figure S9.** Potassium ion (K^+^) leakage from *E. coli* after incubation of TNP or TNW/5 μm under dark and light conditions

**
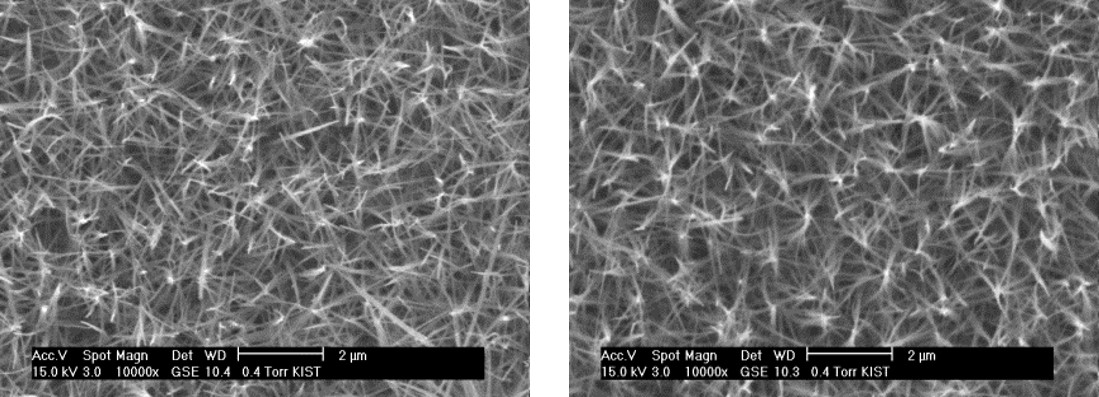
**

**Figure S10.** SEM images of TNW/5 μm recycled after the 5th reaction in the dark (left) and UV light (right).

**Table S3.** Inactivation efficiencies (%)*^a^* of TNW 5/μm and TNP against single cultures and mixed cultures of *E. coli*, *S. aureus*, and MS2 phage*^b^* upon a 30 min exposure.

|  | | | TNW/5 µm | TNP |
| --- | --- | --- | --- | --- |
| Single culture | Dark | *E. coli* | 41.6±7.1 | 3.45±1.3 |
|  |  | *S. aureus* | 46.4±6.3 | 8.29±2.3 |
|  |  | MS2 phage | 74.8±2.9 | 3.13±4.2 |
|  | UV light | *E. coli* | 94.9±0.5 | 59.4±4.8 |
|  |  | *S. aureus* | 99.8±0.1 | 65.9±4.6 |
|  |  | MS2 phage | 100±1.5 | 100±3.1 |
| Mixed culture | Dark | *E. coli* | 38.0±4.8 | 0.55±4.1 |
|  |  | *S. aureus* | 42.9±5.1 | 4.3±1.9 |
|  |  | MS2 phage | 67.9±3.5 | 1.13±1.0 |
|  | UV light | *E. coli* | 79.6±8.8 | 32.5±0.2 |
|  |  | *S. aureus* | 82.2±2.1 | 40.2±1.3 |
|  |  | MS2 phage | 99.1±2.3 | 92.3±2.2 |

*^a^* Values represent means±s.d.s of three independent experiments.

*^b^* Initial concentrations of *E. coli*, *S. aureus*, and MS2 phage are ~10^7^ CFU/ml and ~10^7^ PFU/ml, respectively.
